# Herniation after deep circumflex iliac artery flap: two cases of rare complication

**DOI:** 10.1186/s40902-016-0055-3

**Published:** 2016-02-25

**Authors:** Hee-Sung Kim, Jae-Young Kim, Hyuk Hur, Woong Nam

**Affiliations:** 1 Department of Oral and Maxillofacial Surgery, Yonsei University College of Dentistry, Yonsei-ro 50, Seodaemun-gu, Seoul, 120-752 Korea; 2 Department of Oral and Maxillofacial Surgery, Gangnam Severance Hospital, Yonsei University College of Dentistry, Seoul, Korea; 3 Department of Surgery, Yonsei University College of Medicine, Seoul, Korea; 4 Department of Oral and Maxillofacial Surgery, Oral Cancer Research Institute, Yonsei University College of Dentistry, Yonsei-ro 50, Seodaemun-gu, Seoul, 120-752 Korea

**Keywords:** Deep circumflex iliac artery (DCIA) flap, Hernia, Lichtenstein tension-free hernioplasty, Reconstruction, Bowel obstruction

## Abstract

Herniation after harvesting of deep circumflex iliac artery (DCIA) flap is a known but not a common complication. It occurs about 2.8 to 9 % according to the literatures and can proceed to a more severe complication such as bowel obstruction. There are several factors that exacerbate the risk: surgical factors, operator factor, and patient factors. Surgical factors include large anatomical defect and denervation of related muscles. Operator factor stands for unpunctual suture technique. Patient factors represent obesity, diabetes, pulmonary disease, smoking habits, and so on. Thus, herniation might occur regardless of meticulous suture. Herein, we would like to report two cases of herniation after DCIA flap harvesting and repaired by Lichtenstein tension-free hernioplasty with literature review.

## Background

Deep circumflex iliac artery flap (DCIA) is first introduced by Taylor et al. in 1979 [1]. Since then, it has been used to reconstruct composite defect in the head and neck area. It provides similar contour of mandible and sufficient volume to accommodate implants than other osseous flaps [2]. However, several donor site complications were reported after DCIA flap such as damage of lateral cutaneous femoral nerve, gait disturbance, bowel obstruction, and herniation [3, 4].

Among them, herniation occurs from 2.8 to 9 % of patients and can lead to a more severe condition such as bowel obstruction [5, 6]. It may show various ranges of symptoms from acute to long-standing discomforts after several months or years of operation [7]. Such as a bulge in the groin, pain in the groin that may also include a heavy or dragging sensation, pain followed by tenderness, and nausea, vomiting, or fever caused by bowel obstruction. When patients complain of such abdominal pain, computed tomography (CT) scan is used for confirmation of diagnosis and operation will be performed by various methods including Lichtenstein tension-free technique [8, 9]. When herniation occurs, we can consider several methods like Bassini repair, Shouldice repair, and tension-free hernioplasty. Among them, tension-free hernioplasty is known as a representative method.

Herein, we present two cases of hernia formation after DCIA flap which were treated by Lichtenstein tension-free hernioplasty, cooperated with the Department of Surgery.

## Case presentation

### Case 1

**Fig. 1:**
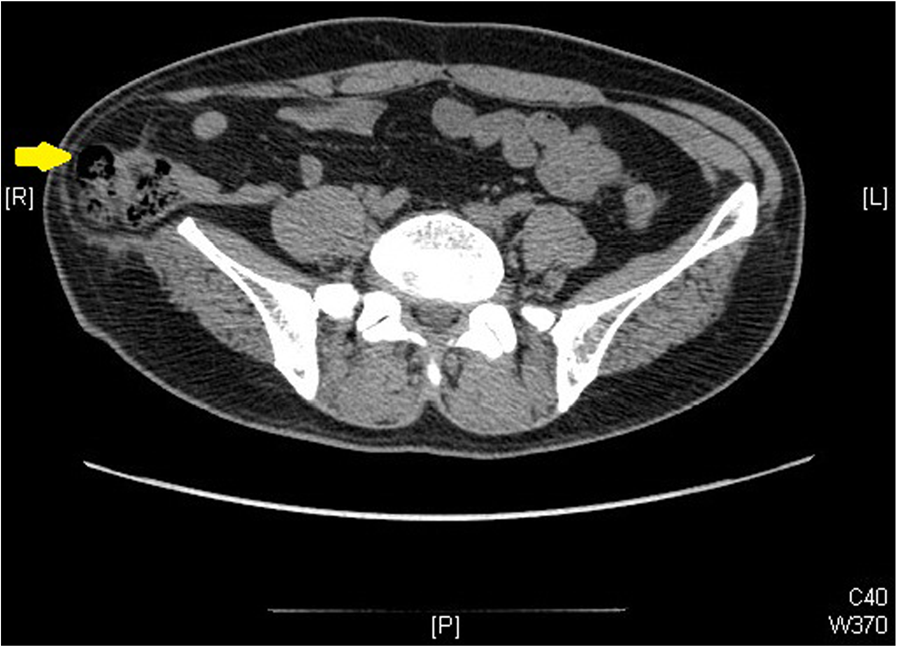
CT image (case 1). Herniation (*arrow*) can be observed through bone defect caused by DCIA flap harvesting. There are no anatomical barriers to avoid herniation

**Fig. 2:**
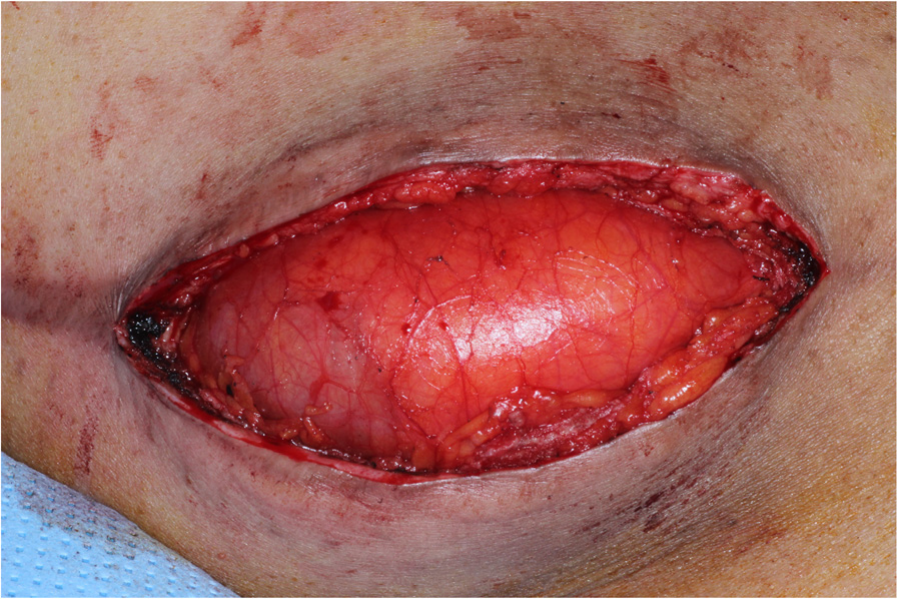
Clinical photograph (case 1). Herniated small bowel and atrophic change of the surrounding muscle and fascia

**Fig. 3:**
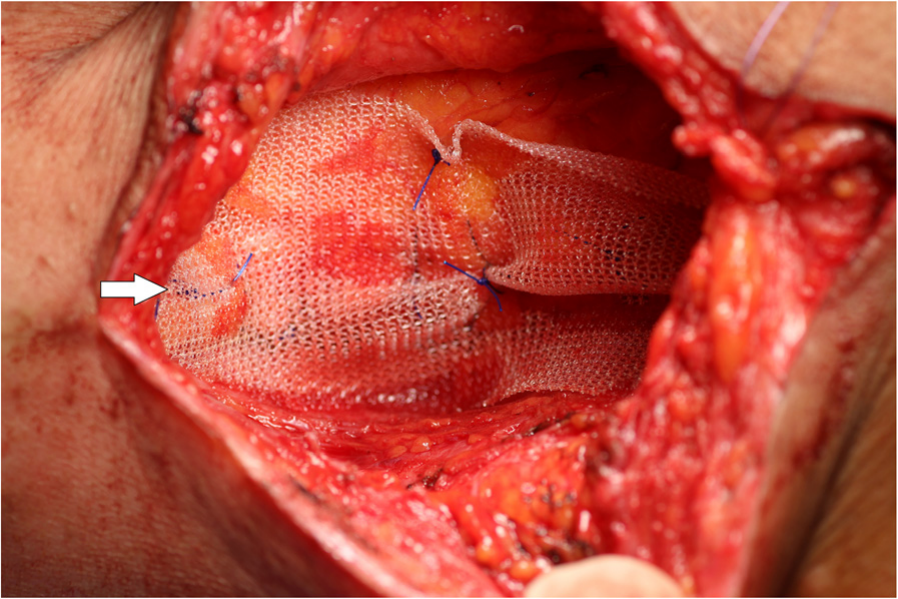
Lichtenstein tension-free hernioplasty. Mesh (*arrow*) is applied over and sutured with surrounding structures

A 53-year-old male visited the Department of Oral and Maxillofacial Surgery at the Dental Hospital. His chief complaint was swelling and pain on left mandibular molar about 5 months ago. There was no specific past medical history. But he was a heavy smoker. He smoked two packs of cigarettes per day during 10 years. His body mass index (BMI) was 27.04 (height 172 cm, body weight 80 kg). Left mandibular second premolar and first molar showed severe mobility accompanied by gingival swelling, pus discharge, and easy bleeding on probing. Incisional biopsy revealed invasive squamous cell carcinoma. Additional imaging studies including magnetic resonance imaging (MRI) and positron emission tomography (PET) were done. Osteolytic lesion and enlarged lymph nodes in the left level II with intense fluorodeoxyglucose (FDG) uptake were observed on the PET image. Thus, operation was performed under general anesthesia as follows: wide excision, segmental mandibulectomy, modified radical neck dissection, and reconstruction of hard tissue defect with DCIA flap harvested from the right side. DCIA flap was harvested with internal oblique muscle. Layer-by-layer suture was carried out precisely to prevent herniation. Unfortunately, however, 2 months after surgery, he visited our department with dull pain on flap harvesting site when palpated. He was referred to Department of Surgery for further evaluation and proper management. He was diagnosed as incisional hernia on the right flank area after CT taking (Fig. 1). Periodic checkup and surgical treatment were planned if herniation becomes worse. After 5 months, he underwent operation because herniation with pain was getting worse. The operation was performed at Department of Surgery. We could observe the herniation of small bowel through the defect formed by DCIA flap harvesting and atrophic change of surrounding muscle and fascia (Fig. 2). Anterior abdominal muscles were sutured to adjacent periosteum with 2-0 Prolene. Mesh (Bard™ mesh, 10 × 14 in., Bard Davol Inc., Warwick, USA) was applied over and sutured with the surrounding structures (Fig. 3). Eight months after herniorrhaphy, he complained of dull pain of the operation site and right lower flank area protrusion. Department of Surgery diagnosed recurrence of herniation, and patient was diagnosed as recurrence of herniation. He underwent muscle approximation with layer-by-layer suture which was the same method used at the previous operation. It has shown a satisfactory result during 7 months to date without any recurrence.

### Case 2

A 52-year-old male underwent segmental mandibulectomy and reconstruction of the defect with DCIA flap due to recurrent invasive squamous cell carcinoma. There was no specific past medical history. But he was a heavy smoker. A 6.5 × 3.0 cm-sized DCIA flap was harvested with internal oblique muscle. Peritoneum was not exposed during flap harvesting. Meticulous suture was performed as usual.

**Fig. 4:**
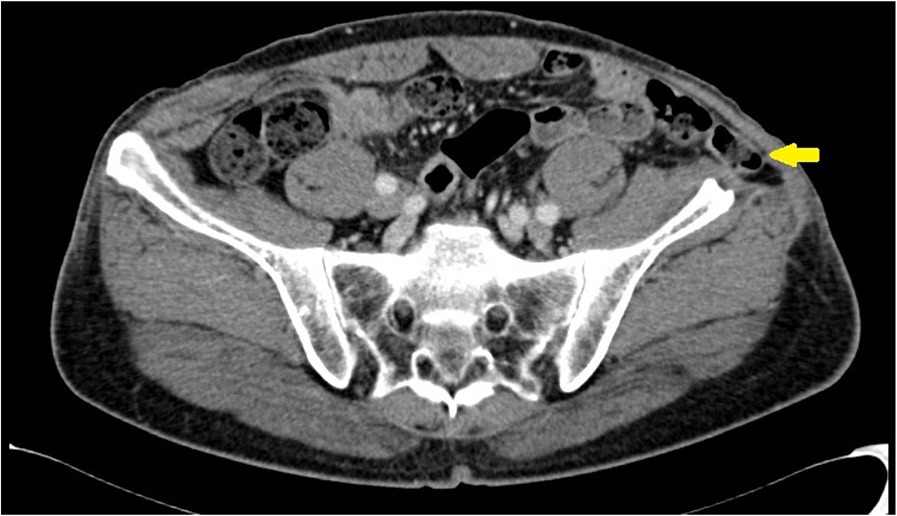
CT image (case 2). Herniation (*arrow*) can be observed through the bone defect caused by DCIA flap harvesting. There are no anatomical barriers to avoid herniation

**Fig. 5:**
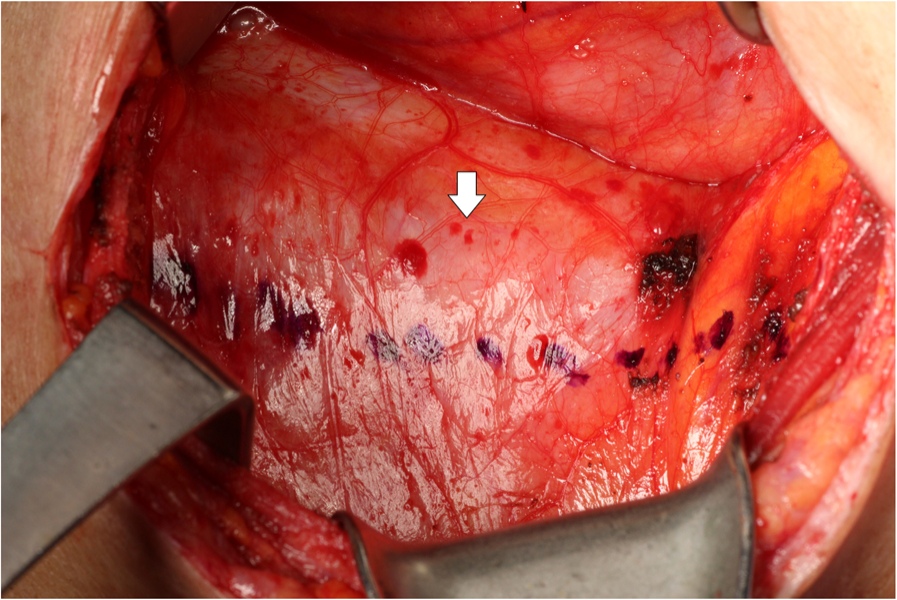
Clinical photograph (case 2). Herniated sac (*arrow*) is identified through the bone defect caused by the previous surgery

After 6 months, the patient complained of dull pain on DCIA flap donor site. We suspected herniation and consulted to Department of Surgery for further evaluation. The patient was diagnosed as incisional ventral hernia. We could find the herniated bowel through iliac bone defect on CT image (Fig. 4). After 4 months (11 August 2014), operation for repair of ventral hernia was performed by general surgeons. Hernia sac was identified on the bony defect area formed by the previous surgery (Fig. 5). The transverse abdominis muscle wall became thin with atrophic change. After excision of herniated sac, the peritoneum was closed by continuous suture. The transverse abdominis muscle was plicated and fixed on iliac crest periosteum to reinforce weakened muscle layer. Then, mesh (Bard™ mesh 10 × 14 in., Bard Davol Inc., Warwick, USA) was applied over the transverse abdominis muscle. Interrupted suture was performed on internal oblique muscle. Finally, we confirmed that the weakened muscle area had disappeared and herniation was properly repaired. Fifteen months have passed until now without any recurrence of herniation.

### Discussion

DCIA flap is a preferable reconstructive method after segmental maxillectomy or mandibulectomy. It is possible to reconstruct the defect area as well as bring functional and esthetic results [10, 11]. The bony stock and height is proper for osteointegration of dental implant and bearing bite force [12]. Reshaping is not necessary during surgery due to its original anatomical shape. Hairless skin on the groin area is suitable for re-coverage of intraoral mucosa [11]. Rogers et al. reported better quality of life and lesser donor site morbidity when DCIA flap was used compared with fibula-free flap [13].

Although DCIA flap has several advantages, donor site morbidities such as gait disturbance, lateral femoral cutaneous nerve damage, and herniation make it difficult for the patients as well as the operators. Especially, occurrence of herniation after several months or years of operation makes it difficult for the operator to manage the patient as he needs additional surgery [7]. According to the literature, the incidence is reported from 2.8 to 9 % [5, 6]. The patients complain acute abdominal discomfort or long-term pain and an abdominal mass showing internal noise at auscultation as well as palpation [8]. CT image is used for definite diagnosis [8]. In these cases, herniation was also developed at 2 and 6 months after surgery with dull pain on the donor site. Sometimes, pelvic bone fracture can occur after harvesting of DCIA flap or iliac bone graft. It is especially true in case of osteopenic conditions and operations including anterior iliac crest [14] .At first, we also suspected fracture of pelvic bone because of their obesity which has adverse effects on healing process. Especially, it was more suspicious in case 1, because his heavy weight could bring more pressure on pelvic bone and only 2 months had passed after surgery. However, the impression was herniation due to several reasons. They were not osteopenic conditions, and anterior-superior iliac spine was preserved more than 3 cm [14]. The patient in case 1 had seldom walked during 2 months because he underwent several other operations. After CT taking, definite diagnosis as herniation was made.

A number of reasons were suggested for herniation at a donor site. First, anatomical defect is related. DCIA flap is harvested with the surrounding muscles to maintain blood supply. Defect of the surrounding muscles causes loss of anatomical barrier, which brings herniation when managed improperly [15]. The flap must include the skin, external oblique, internal oblique, and transverse abdominis muscle to keep the perforators. This makes bigger defect size and raises the possibility for herniation. Forrest et al. reported the incidence was 12 % (7/59) among osteocutaneous flaps, whereas the incidence was 4 % (1/23) among osseous flaps [16]. Second, we consider the general conditions of the patient such as history of chronic obstructive pulmonary disease, obesity, diabetes, gender, constipation, and smoking habits [4, 17]. Especially, obese patients have greater risk of incisional hernia due to increased intra-abdominal pressure [18]. Other perioperative factors including inadequate analgesia, vomiting, and postoperative pneumonia are also considered [4]. Furthermore, the risk of herniation may be exacerbated by denervation of rectus muscle [3]. Internal oblique muscle harvesting may bring this denervation through interruption of motor nerve supply [19]. We can prevent this denervation with precise dissection of the nerves, which lie between the internal oblique and transverse abdominal muscles [19]. In these cases, we consider that the primary reason is improper management of the surrounding muscles. Besides, we take harvesting of the internal oblique muscle, obesity, and previous heavy smoking habits into consideration.

Surgical techniques and materials have been proposed to prevent or minimize hernia formation. First of all, surgeons ought to make an effort to restructure the abdominal wall. Meticulous suturing of the transversalis fascia to the iliacus fascia, and all three layers of the abdominal muscles to the tensor fascia latae and gluteus medius are necessary [16]. Apart from the primary closure technique, Halsnad et al. suggested the use of titanium plate for primary reconstruction of the osseous defect [7]. In 2007, Iqbal et al. reported insertion of titanium coils (Protack™, US Surgical Corp.) to attach Prolene (polypropylene) mesh into the cancellous and cortical bone of the donor site [15]. Despite of these efforts, however, herniation through DCIA flap occurs [4]. We can consider various open surgery techniques such as Bassini repair, Shouldice repair, and tension-free hernioplasty when herniation occurs [20]. Among them, Lichtenstein tension-free hernioplasty is one of the representative methods for hernia repair using polypropylene mesh [21]. It was developed in 1984 and was improved through 1988 [22]. Although some disadvantages were reported such as increasing stiffness of the abdominal wall and abdominal pressure, American College of Surgeons considers this method as a gold standard in hernia repair [22–24].

## Conclusions

Herniation after DCIA flap is a known complication but uncommon. Precise primary suture is always important to prevent herniation. Especially, it is more so when the patient is obese, a heavy smoker, and when the flap contains more muscles including internal oblique muscles. Also, careful observation and follow-up of the donor site, which is the groin area, should be done as well at the recipient site after DCIA flap. If pain or bulging is observed, patient should be referred to the Department of Surgery for evaluation on hernia. And if hernia occurs, repair using Lichtenstein tension-free hernioplasty should be performed at a proper period.

## Consent

Written informed consent was obtained from the patient for publication of this case report and any accompanying images. A copy of the written consent is available for review by the Editor-in-Chief of this journal.
